# Multicenter Clinical Evaluation of the Revogene Strep A Molecular Assay for Detection of Streptococcus pyogenes from Throat Swab Specimens

**DOI:** 10.1128/JCM.01775-19

**Published:** 2020-06-24

**Authors:** D. Banerjee, J. Michael, B. Schmitt, H. Salimnia, N. Mhaissen, D. M. Goldfarb, P. Lachance, M. L. Faron, T. Aufderheide, N. Ledeboer, A. Weissfeld, R. Selvarangan

**Affiliations:** a Children’s Mercy Hospital, Kansas City, Missouri, USA; b Indiana University School of Medicine, Indianapolis, Indiana, USA; c Wayne State University School of Medicine, Detroit, Michigan, USA; d Valley Children’s Hospital, Fresno, California, USA; e BC Children’s and Women’s Hospital, Vancouver, British Columbia, Canada; f Centre de Recherche Saint-Louis, Quebec City, Canada; g Medical College of Wisconsin, Milwaukee, Wisconsin, USA; h Microbiology Specialists Inc., Houston, Texas, USA; bioMérieux

**Keywords:** clinical trial, group A streptococcus, molecular assay, multicenter

## Abstract

Group A streptococcus (GAS) species cause bacterial pharyngitis in both adults and children. Early and accurate diagnosis of GAS is important for appropriate antibiotic therapy to prevent GAS sequalae. The Revogene Strep A molecular assay (Meridian Bioscience Canada Inc, Quebec City, QC, Canada) is an automated real-time PCR assay for GAS detection from throat swab specimens within approximately 70 min. This multicenter prospective study evaluated the performance of the Revogene Strep A molecular assay compared to that of bacterial culture. Dual throat swab specimens in either liquid Amies or Stuart medium were collected from eligible subjects (pediatric population and adults) enrolled across 7 sites (USA and Canada). Revogene Strep A and reference testing was performed within 7 days and 48 h of sample collection, respectively. Of the 604 evaluable specimens, GAS was detected in 154 (25.5%) samples by the reference method and in 175 (29%) samples by the Revogene Strep A assay. Revogene Strep A assay sensitivity and specificity were reported to be 98.1% (95% confidence interval [CI], 94.4 to 99.3) and 94.7% (95% CI, 92.2 to 96.4), respectively. The positive predictive value was 86.3% (95% CI, 80.4 to 90.6), negative predictive value was 99.3% (95% CI, 98.0 to 99.8) with a 1.0% invalid rate. Discrepant analysis with alternative PCR/bidirectional sequencing was performed for 24 false-positive (FP) and 3 false-negative (FN) specimens. Concordant results were reported for 17 (FP only) of 27 discordant specimens. The Revogene Strep A assay had high sensitivity and specificity for GAS detection and provides a faster alternative for GAS diagnosis.

## Abstract (precis)

Group A streptococcus (GAS) species cause bacterial pharyngitis in both adults and children. Early and accurate diagnosis of GAS is important for appropriate antibiotic therapy to prevent GAS sequalae. The Revogene Strep A molecular assay (Meridian Bioscience Canada Inc, Quebec City, QC, Canada) is an automated real-time PCR assay for GAS detection from throat swab specimens within approximately 70 min. This multicenter prospective study evaluated the performance of the Revogene Strep A molecular assay compared to that of bacterial culture.

## INTRODUCTION

Acute pharyngitis, although primarily of viral etiology, is also caused by Streptococcus pyogenes, a member of the group A streptococcus (GAS) by Lancefield serogrouping and is more commonly isolated in children between 3 and 15 years of age (1). GAS species are isolated in up to 37% of sore throat episodes in children older than 5 years (2, 3), whereas their prevalence in adult patients is estimated to be in the range of 5% to 10% (4). GAS presents with a wide spectrum of diseases that ranges from uncomplicated pharyngitis and skin infections to severe sepsis, toxic shock syndrome, and necrotizing fasciitis. Nonsuppurative sequalae of GAS infection, mainly acute glomerulonephritis (AGN) and acute rheumatic fever (ARF), account for significant cause of global morbidity and mortality, with a particular impact in resource-limited settings (5, 6). Annually, 500,000 people worldwide develop ARF, with 223,000 deaths reported each year that are directly attributed to ARF or to subsequent rheumatic heart disease (7, 8). However, in more affluent countries, the majority of S. pyogenes-associated deaths are associated with invasive disease (5). Accurate diagnosis and prompt initiation of antibiotic therapy are essential for treatment and prevention of sequalae.

Current guidelines for diagnosis of GAS pharyngitis in children and adolescents recommend the testing of throat swab specimens by a rapid antigen detection test (RADT) with a follow-up of RADT-negative specimens by culture (9). Routine backup with culture is not recommended in adults with a negative RADT result because of the low incidence of sequelae of pharyngitis in this population (9). RADT assays are easy to perform and have a rapid turnaround time (TAT; <15 min) with high specificity (95%). However, the relatively low sensitivity (70 to 90%) of RADTs prompts the need for a backup culture in the RADT-negative specimens from children and young adults (3, 10, 11). Bacterial culture, the gold standard method, is both highly sensitive and specific (90 to 95%), but is labor-intensive and requires a minimum of 24 to 48 h for reporting a positive result (12). More recently, nucleic acid amplification testing (NAAT) assays have become popular due to their enhanced sensitivity and specificity and faster TAT (13). Implementation of PCR as a stand-alone diagnostic test for GAS in a clinical setting has led to a significant increase in positive laboratory test results (14). Prompt and appropriate methods of testing can help lower the incidence of inappropriate antibiotic use, mainly in patients with viral pharyngitis. However, correct patient selection is important to limit low levels of GAS detection by highly sensitive PCRs in asymptomatic carriers (15). The use of molecular assays for detecting GAS pharyngitis could be an important addition to the guidelines.

The recently Food and Drug Administration (FDA)-cleared Revogene Strep A molecular assay, performed on the Revogene instrument, is an automated, qualitative, moderately complex *in vitro* diagnostic test that detects GAS nucleic acids directly from throat swab specimens by real-time PCR (RT-PCR). The Revogene instrument is capable of automated cell lysis, dilution of nucleic acids, automated amplification, and detection of target nucleic acid sequences in as few as 42 min for GAS-positive and 70 min for GAS-negative samples. In this multicenter study, we evaluated the clinical performance of the Revogene Strep A assay in detecting GAS from throat swab specimens prospectively collected from subjects with pharyngitis.

## MATERIALS AND METHODS

### Study design.

This prospective multicenter study was conducted between February 2018 and July 2018 at 7 geographically diverse sites across the United States (Missouri, California, Michigan, Indiana, and Wisconsin) and Canada (British Columbia and Quebec). There was one reference center in each country, namely Texas in the United States and British Columbia in Canada. Throat swab specimens were collected from subjects in accordance with site-specific institutional review board-approved protocols. Inclusion criteria were (i) patients presenting with signs and symptoms of pharyngitis, (ii) informed consent obtained from subject or legal guardian, (iii) patient was older than 2 years of age, and (iv) patient provided a dual swab in either liquid Stuart or liquid Amies transport medium. Exclusion criteria were (i) use of antibiotics within 7 days prior to specimen collection and (ii) any specimen not compliant with the study protocol.

### Specimens.

Specimens from subjects >2 years of age with signs and symptoms of pharyngitis were enrolled. Demographic information about subjects from different sites is listed in Table 1. Dual throat swab specimens were collected using Copan (Italy) or BD swabs. The swab heads were rubbed together for uniform sampling; one swab was broken off and placed in the Sample buffer tube (SBT) to test with the Revogene Strep A assay at the collection center, while the second swab was placed in transport medium (liquid Amies for Quebec, Michigan, Indiana, and Missouri sites and liquid Stuart medium for British Columbia, Wisconsin, and California sites) and shipped to the reference centers for reference method testing. Samples for reference testing were sent to the respective Canadian or American reference centers at 2° to 8°C within 48 h of collection. Culture for retrieval of *Streptococcus* was initiated within 72 h of collection. The specimen in SBT was stored at 2° to 8°C and tested at the collection sites within 7 days on the Revogene Strep A assay. After obtaining a valid result or following a single repeat test, an aliquot of SBT was prepared and stored at −20°C or colder and was shipped to the sponsor periodically.

**TABLE 1:** Site-wide distribution of specimens and GAS prevalence by reference culture

Patient characteristic	No. (%) of specimens from site:							
	1 (*n* = 69)	2 (*n* = 67)	3 (*n* = 42)	4 (*n* = 123)	5 (*n* = 178)	6 (*n* = 49)	7 (*n* = 76)	Total
Age								
24 mo–12 yrs	53 (76.8)	0 (0.0)	0 (0.0)	114 (92.7)	12 (6.7)	40 (81.6)	59 (77.6)	278 (46)
13–21 yrs	15 (21.7)	4 (5.9)	5 (11.9)	7 (5.7)	24 (13.5)	9 (18.4)	16 (21)	80 (13.2)
22–64 yrs	1 (1.5)	62 (92.5)	32 (76.2)	1 (0.8)	138 (77.5)	0 (0.0)	1 (1.4)	235 (38.9)
>65 yrs	0 (0.0)	1 (1.6)	5 (11.9)	1 (0.08)	4 (2.3)	0 (0.0)	0 (0.0)	11 (1.8)
Ethnicity								
White	0 (0.0)	60 (89.5)	21 (50)	18 (14.6)	59 (33.1)	15 (30.6)	15 (19.7)	188 (31.1)
Hispanic	0 (0.0)	4 (5.9)	0 (0.0)	8 (6.5)	21 (11.8)	10 (20.4)	53 (69.7)	96 (15.9)
Black/African American	0 (0.0)	1 (1.6)	20 (47.6)	93 (75.6)	89 (50)	24 (49)	3 (3.9)	230 (37.9)
Asian	0 (0.0)	1 (1.6)	1 (2.4)	1 (0.8)	0 (0.0)	0 (0.0)	2 (2.6)	5 (0.8)
Unknown	69 (100)	1 (1.6)	0 (0.0)	3 (2.4)	9 (5.1)	0 (0.0)	3 (3.9)	85 (14.1)
GAS culture result								
Positive	16 (23.2)	18 (26.9)	2 (4.8)	59 (48)	30 (16.9)	13 (26.5)	16 (21.1)	154 (25.5)
Negative	53 (76.8)	49 (73.1)	40 (95.2)	64 (52)	148 (24.5)	36 (73.5)	60 (78.9)	450 (74.5)

### Revogene Strep A assay.

The broken swab in SBT was processed on the Revogene within 7 days of collection. A set of environmental controls were run at the beginning of the study, following which external positive and negative controls were run each day samples were tested by the Revogene Strep A assay. Sample testing involved adding a designated amount of SBT (120 μl to 200 μl) to the disposable microfluidic cartridges (PIE), which were then loaded onto the Revogene instrument. Up to 8 samples could be tested on the Revogene simultaneously. The amplified products were detected in real time using target-specific TaqMan chemistry-based probes. On completion of a run, the results were computed by the system from measured fluorescent signals and embedded calculation algorithms. Samples that initially produced a nonreportable result (unresolved, indeterminate or external control [EC] failure) were repeat tested following instructions from the package insert. Samples with a reportable (positive, negative) result upon repeat testing were included in the data analyses.

### Reference testing.

Samples for reference testing were cultured on blood agar plates (BAP) and incubated for 24 to 48 h at 35° to 37°C under 5% CO_2_ or anaerobic conditions. Colonies morphologically resembling Streptococcus pyogenes (GAS) were isolated on a new BAP and reincubated for 24 h under similar conditions before proceeding to identification tests. Identification was confirmed by catalase test (nonreactive), Gram staining (Gram-positive cocci in chains), presence of beta hemolysis, and a GAS-specific latex agglutination test. Isolated colonies identified as GAS by latex were distributed into 2 aliquots and frozen for additional testing at the reference centers. Frozen isolated colonies were thawed, plated on BAP, and further characterized using matrix-assisted laser desorption ionization-time of flight mass spectroscopy (MALDI-TOF MS) identification at the U.S. reference center. The Canadian reference center processed samples, including culture and colony identification, following which the frozen aliquots were shipped to the U.S. reference center for MALDI-TOF MS.

### Discordant analysis.

Specimens with discordant findings between the Revogene Strep A assay and reference method were further analyzed by alternative PCR/bidirectional sequencing. Sample buffer leftover from the Revogene Strep A test was frozen without the swab and sent to the Centre de Recherche en Infectiologie, where the sample buffer underwent mechanical and thermal lysis and was mixed with the PCR reagents. PCR was performed on the CFX96 (Bio-Rad) instrument, targeting a proprietary GAS-specific gene other than the target used in Revogene Strep A assay. If PCR led to an amplification, the amplicon was purified and sent for bidirectional sequencing.

### Statistical analysis.

**(i) Sample size calculation.** With the average prevalence of Streptococcus pyogenes in clinical specimens at 20% in North America, at least 600 samples needed be enrolled to achieve the study objective of 120 samples positive for GAS by reference method.

**(ii) Descriptive analysis.** Overall GAS prevalence was determined by each site and age group.

**(iii) Analytical statistics.** Revogene data were analyzed using software v4.0.10 and assay definition file v0.2.1. Performance characteristics (sensitivity, specificity, positive predictive value [PPV], and negative predictive value [NPV]) of the Revogene Strep A assay in comparison to those of the reference method were calculated using two-by-two tables. Data are presented as value ± 95% confidence intervals (CI). Confidence intervals were determined using the Wilson scoring method. The invalid rate was determined based on samples that resulted as either “unresolved” (due to sample inhibitors or failure of internal positive control to amplify) or “indeterminate” (instrument failure).

## RESULTS

A total of 767 unique throat swab specimens were collected in the study period across 7 sites, of which 163 (21.3%) specimens were excluded from performance calculations. Exclusions were primarily due to (i) deviation from reference method culture protocol (100, 61.3%), (ii) erroneous PCR results (due to external control failure, deviations from package insert instructions, or indeterminate/unresolved results after repeat testing) (13, 8.0%), (iii) deviations based on transport and storage time/conditions (13, 7.9%), (iv) patients missing PCR or reference method results (13, 8.0%), (v) specimens collected outside clinical approval (11, 6.7%), (vi) patients younger than 24 months old (6, 3.7%), (vii) patients using antibiotics (3, 1.8%), (viii) patients enrolled after the study period (2, 1.2%), and (ix) duplicate specimens (2, 1.2%). Overall, 604 evaluable specimens were included, with individual sites providing between 7% and 29.5% of the total study samples. The majority of the enrolled subjects were within 2 to 12 years old (278, 46%), followed by patients ranging between 22 to 64 years (235, 38.9%). Detailed patient demographic information is provided in Table 1.

Of the 604 unique samples that were evaluated, 175 (29.0%) were reported as positive by the Revogene Strep A assay. Eleven specimens yielded either “unresolved” (*n *= 3) or “indeterminate” (*n *= 8) results in the initial run, demonstrating an initial invalid rate of 1.8% (11/610). After repeat testing, only 6 specimens yielded “unresolved” (*n* = 1) or “Indeterminate” (*n* = 5) results, resulting in a final invalid rate of 1.0% (6/610). Reference culture tested positive for 154 samples (25.5%). Site-wide GAS prevalence by reference culture is demonstrated in Table 1. The Revogene Strep A assay showed a sensitivity and specificity of 98.1% (95% CI, 94.4 to 99.3) and 94.7% (95% CI, 92.2 to 96.4), respectively (Table 2). The PPV was reported to be 86.3 (95% CI, 80.4 to 90.6), while the NPV was 99.3 (95% CI, 98.0 to 99.8). Three (3) specimens were false negative (FN) and 24 specimens were false positive (FP) by Revogene Strep A assay compared to reference culture.

**TABLE 2:** Clinical performance of the Revogene Strep A assay^*a*^

Revogene Strep A assay result	Bacterial culture plus MALDI-TOF MS results (no.)		
	Positive	Negative	Total
Positive	151	24	175
Negative	3	426	429
Total	154	450	604

a Sensitivity and specificity of the Revogene Strep A assay were 98.1 (95% CI, 94.4 to 99.3) and 94.7 (95% CI, 92.2 to 96.4), respectively, and the PPV and NPV were 86.3 (95% CI, 80.4 to 90.6) and 99.3 (95% CI, 98.0 to 99.8), respectively.

Discrepant analysis was performed on 27 specimens by retesting a frozen aliquot from the sample buffer tube at a third-party facility (Centre de Recherche en Infectiologie de Quebec) by an alternative PCR assay followed by bidirectional sequencing (Table 3). Results were reported as positive, negative, or inconclusive (sequence could not be reported due to poor quality). Of the 24 FP samples, the majority were from subjects aged 24 months to 12 years old (14/24, 58.3%), followed by subjects who were 22 to 64 years old (7/24, 29.2%). In total, 17/24 specimens (70.8%) that were FP by Revogene Strep A assay were positive for GAS by discrepant testing, while 1/24 provided inconclusive result. A total of 6/24 FP results were negative for GAS. One of the 3 specimens (33.3%) that were FN with Revogene Strep A assay was positive for GAS by discrepant testing. However, the remaining 2 specimens remained inconclusive.

**TABLE 3:** Discrepant analysis of Revogene Strep A assay compared to culture

No. of samples	Revogene assay result	Culture plus MALDI-TOF MS result	Result type^*a*^	PCR result/sequencing assay
17	Positive	Negative	FP	Positive
6	Positive	Negative	FP	Negative
1	Positive	Negative	FP	Inconclusive
2	Negative	Positive	FN	Inconclusive
1	Negative	Positive	FN	Positive

a FP, false positive; FN, false negative.

## DISCUSSION

The gold standard for diagnosis of GAS pharyngitis is the isolation of bacteria on a 48-h culture (1). However, due to the associated time lag between sample collection and test results and the labor intensiveness of the procedures involved, faster and easier alternatives such as RADTs are more commonly employed for detecting GAS. Most of these assays report a high specificity, ruling out the need for a backup culture in a positive RADT result (12, 13). However, the currently available RADTs lack sensitivity, potentially leading to missed diagnosis of true GAS infection in patients (11, 16, 17). The need for highly sensitive and rapid assays to compete against the more time-consuming culture methods and less sensitive RADTs led to the development of molecular assays. These assays demonstrate superior sensitivity compared to RADTs without a loss of specificity, and they eliminate the need for culture confirmation, making them an attractive option for laboratory diagnosis of GAS (13, 14, 18).

Several FDA-approved nucleic acid amplification assays are available and include (Clinical Laboratory Improvement Amendments) CLIA waived and CLIA moderate complexity tests (13). These assays are based on a number of different principles that range from real-time PCR detection to loop-mediated isothermal amplification (LAMP) in the *illumi*gene assay (Meridian BioScience) and isothermal helicase-dependent amplification (HDA) in the Solana GAS and Solana Strep Complete assays (Quidel Corp.). The Simplexa Group A Strep direct kit (Diasorin Molecular), Cobas Strep A assay (Roche Diagnostics), Aries Group A Strep assay (Luminex Corporation), Lyra Direct Strep (Quidel Corporation), and Xpert Xpress Strep A (Cepheid) are real-time PCR assays that target different conserved regions of the GAS genome (*sdaB*, *speB*, *spy1258*, or *comx1.1* genes) (13). Previous studies on respective platforms have demonstrated high sensitivities and specificities (13, 19–24). The ID Now Strep A 2 assay (Abbott lab) is based on the principle of isothermal nicking enzyme amplification reaction (NEAR) (18, 25) and has comparable performance. In addition to the high diagnostic performance, the Xpert Xpress, Cobas Strep A, and ID Now Strep A 2 assays are point-of-care systems that are easy to perform and exhibit a TAT ranging between 6 and 24 min, which has made them increasingly popular.

This is the first study to evaluate the performance characteristics of the recently developed Revogene Strep A assay compared to reference culture and MALDI-TOF MS identification. Results from our study demonstrated comparable sensitivity and specificity to other molecular assays (>94% for both) for detecting GAS in throat swabs from patients with acute pharyngitis. Of the 3 specimens that tested negative (FN) on Revogene Strep assay compared to culture, one was negative by alternative PCR/bidirectional sequencing assay as well, and 2 specimens were inconclusive, suggesting the presence of a potential inhibitor in the sample itself that was interfering with the amplification. Of the 24 apparent false positives, 17/24 tested positive by alternative methods, which might be due to the presence of GAS nucleic acids only (in the absence of viable organisms) from patients with resolving infections or who are already undergoing antimicrobial therapy (26). Furthermore, the Revogene Strep A assay, being highly sensitive, might detect very low levels of bacterial load in samples that were otherwise negative by culture (14). In the analytical assessments in this study, 599/610 samples gave valid results in the initial run, and 5/11 samples with invalid initial results demonstrated valid outcomes on repeat testing.

In addition to high sensitivity and specificity and low error/invalid rates, the Revogene Strep A assay is also easy to perform and requires minimum hands-on time (approximately 3 min per sample). Revogene TAT is approximately 70 min for negative and as short as 42 min for positive samples, thereby enabling physicians to initiate appropriate treatment promptly. Simultaneous processing and testing of up to 8 samples provides the flexibility to the operators for either batch testing or sequential determinations. Testing can also be performed multiple times a day in laboratories with high throughput. Accurate results due to the high sensitivity/specificity of the assay bypass the need for culture confirmation and could be used as the standalone confirmatory diagnostic method for diagnosing GAS pharyngitis.

Overall, the Revogene Strep A assay is highly sensitive and specific, easy to perform, and provides reproducible results among different users in different clinical settings. These aspects make it a useful diagnostic tool and an attractive alternative to other rapid diagnostic tests for GAS pharyngitis.
